# Optimizing size selectivity and catch patterns for hake (*Merluccius merluccius*) and blue whiting (*Micromesistius poutassou*) by combining square mesh panel and codend designs

**DOI:** 10.1371/journal.pone.0262602

**Published:** 2022-01-20

**Authors:** Elsa Cuende, Manu Sistiaga, Bent Herrmann, Luis Arregi

**Affiliations:** 1 Marine Research, AZTI Basque Research and Technology Alliance (BRTA), Sukarrieta, Bizkaia, Spain; 2 Fish Capture Division, Institute of Marine Research, Bergen, Norway; 3 Department of Marine Technology, Norwegian University of Science and Technology, Trondheim, Norway; 4 Fisheries and New Biomarine Industry, SINTEF Ocean, Fishing Gear Technology, Hirtshals, Denmark; 5 The Norwegian College of Fishery Science, UiT The Arctic University of Norway, Tromsø, Norway; 6 Fisheries Technology, DTU Aqua, Denmark Technical University, Hirtshals, Denmark; Swedish University of Agricultural Science, SWEDEN

## Abstract

Gear modifications in fisheries are usually implemented to obtain catch patterns that meet management objectives. In the Basque bottom trawl fishery, gear regulations include the use of a square mesh panel (SMP) placed at the top panel of the extension piece of the trawl to supplement diamond mesh codend selectivity. However, the catch patterns obtained with this combination have raised concern among scientists and authorities. This study combines new data on different SMP and codend designs with existing data from the literature to produce new results that are applied to predict the size selectivity and catch patterns of different gear combinations for a variety of fishing scenarios. A systematic approach based on the concept of treatment trees was outlined and applied to depict the effect of individual and combined gear design changes on size selectivity and catch patterns for hake (*Merluccius merluccius*) and blue whiting (*Micromesistius poutassou*). This approach led to identification of the gear combination with the most appropriate exploitation pattern for these two species and improved the readability and interpretation of selectivity results. The results demonstrated that changes both in SMP and, especially, codend designs have a significant effect on hake and blue whiting size selectivity and catch patterns. Therefore, we believe that further research should prioritize codend size selectivity, and additional selection devices may be added once codend designs with good selective properties are achieved.

## Introduction

Reducing the capture of non-target species and undersized individuals of commercial species is one of the major challenges of fisheries management [1, 2]. In the trawl fisheries of the European Union (EU), considerable effort has been devoted in recent years to reduce discards and comply with the recently reformed Common Fisheries Policy (CFP). The strategies used to meet the new management objectives include the increased use of bycatch, a decrease or relocation of fishing effort and the application of more selective fishing gears [3]. Following the last of these strategies, extensive research has been conducted on towed fishing gears, especially aiming at the development of alternative gear designs to improve catch patterns (i.e., the composition of species and sizes in the catch) in specific fisheries (e.g., [4, 5]).

In several multispecies trawl fisheries, the applied gear designs have been modified to meet changes in management objectives such as quota availability, capture prohibition or discard bans [6–8]. For example, in the multispecies bottom trawl fishery of northern Spain (Basque Country), several fishing regulations were implemented in recent decades to stimulate the recovery of hake (*Merluccius merluccius*) [9–12]. In 2006, a 2 m long, 1 m wide, 100 mm mesh size square mesh panel (SMP) positioned in the upper panel of the trawl’s extension piece was introduced in the regulation to supplement diamond mesh codend selectivity [13]. Combinations of SMPs and diamond mesh codends have been widely used in crustacean trawl fisheries (e.g., [14, 15]) because they can support the release of undersized roundfish while preventing the loss of crustaceans such as *Nephrops*, which usually enter the trawl closer to the lower netting panel [16, 17]. However, in several fish directed fisheries the performance of those gear designs has been unsatisfactory regarding reduction in captures of undersized fish of commercial species [18–20].

The release of fish through SMPs can be more problematic than through sorting grids or codend meshes because SMPs function by relying on fishes’ swimming ability and active contact with the SMP [21–24]. Some gear has been designed with the aim of improving fish contact with the SMP (e.g. [21, 25]), but low contact rates remain a problem. Contact probability can vary between species depending on SMP size and position in the trawl [21, 26]. Cuende et al. [24] showed that the release efficiency of hake through an SMP placed in the bottom panel of the extension piece was significantly higher than that of a larger SMP placed in the top panel of the extension piece, while for blue whiting (*Micromesistius poutassou*) the opposite result was achieved.

Codends can also be modified in multiple ways to affect species and size catch patterns. Mesh size, shape, twine material, twine thickness and codend circumference influence species and size selectivity, both due to differences in behavior and the fish’s ability to physically penetrate the codend meshes [27–29]. In bottom trawls, diamond meshes in the codend are normally only partially open and do not support the release of fish sizes that theoretically could escape through them at higher opening angles [30–32]. To mitigate this problem, some fisheries have implemented the use of codends entirely or partly constructed from square meshes [33], which keep an open shape during trawling [30, 34]. Different studies have demonstrated that compared to diamond mesh codends, square mesh codends can reduce discards, maintaining target catch efficiency [35, 36].

Individually, both SMPs and codends have limitations regarding size selection and consequently on obtainable catch patterns. In addition, the literature covering the size selectivity potential of different SMP and codend designs typically tests only a few gear types, partly for logistic reasons and partly to ensure that there are sufficient hauls to estimate the selection of each gear with reasonable precision. However, studies combining results from previous research have become more common in this field in recent years [16, 37–39], and have proven to be a suitable tool for exploring a broad range of selective gear options for use in a fishery without the time and cost outlay associated to experimental trials [16, 38, 40, 41].

In this study, we aim at identifying which SMP and codend design combination leads to the best catch patterns for hake and blue whiting. For this purpose, the selective properties of different SMPs and codend designs were estimated individually so that the selectivity of different SMP and codend combinations for different population scenarios could later be modeled. These two species are usually captured together by the different trawl fisheries operating in the Bay of Biscay and their condition as target or bycatch species varies depending on the fishery, quota availability and market preferences [7]. Thus, flexibility for the size selective properties of the gear for these species is required. To provide detailed information about the contribution of different SMP and codend designs to the overall size selectivity and catch pattern of the species studied, a systematic approach based on treatment trees was used. Treatment trees tool uses a tree-like structure to depict the effect of different treatments and their consequences using the same procedure as decision trees [42–44]. The growing need to depict several results systematically has recently encouraged the use of this approach in different scientific fields (e.g. [42–44]), including research on fishing gear [45, 46].

Therefore, this study was designed to answer the following research questions:

1. Considering the different designs investigated, what is the optimal SMP and codend combination with respect to size selectivity and catch patterns for hake and blue whiting?
2. Is the use of treatment trees appropriate for investigating and illustrating the effect of multiple gear changes on selectivity and catch patterns systematically and comprehensively?

## Materials and methods

### SMP and codend designs

**Fig 1:**
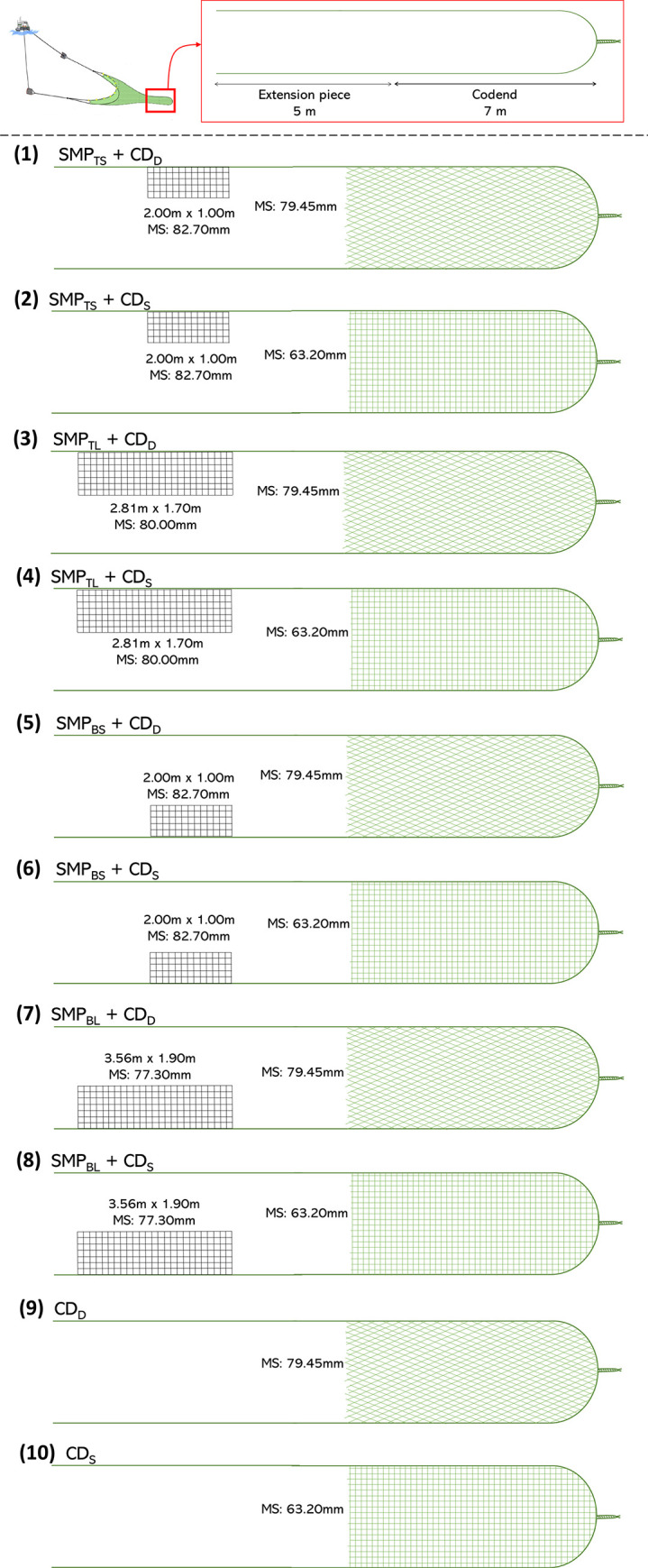
Ten trawl gear configurations included in the study that resulted from combining different SMP and codend designs considered. MS: mesh size.

This study considered trawl gears with different SMP and codend designs to improve size selectivity for hake and blue whiting. Regarding SMP, the following designs were considered: (i) small SMP located at the top panel of the extension piece (SMP_TS_), which is included in current regulation of the fishery; (ii) large SMP located at the top panel of the extension piece (SMP_TL_); (iii) small SMP located at the bottom panel of the extension piece (SMP_BS_); (iv) large SMP located at the bottom panel of the extension piece (SMP_BL_) and (v) absence of SMP in the trawl. Earlier studies showed that the SMP_TS_ performs unsatisfactorily for some species due to lack of contact between fish and the SMP_TS_ [19, 23]. We therefore considered different SMP designs meant to optimize the release efficiency of hake and blue whiting. The second design considered was a larger SMP placed at the upper panel, potentially increasing the chance for contact with the panel. Underwater observations in earlier studies showed that hake prefer swimming close to the lower panel [19, 23, 47], whereas blue whiting has an erratic behavior in the SMP area, swimming quickly either towards the SMP or the codend [23]. Thus, the third and fourth designs consisted respectively of the small SMP in (i) and a larger-size panel placed at the bottom panel of the trawl. Considering that hake individuals, besides entering the trawl close to the lower panel, do not actively swim inside it [23], we tested a SMP_BL_ that was bigger than SMP_TL_ in case (iv). The aim was to potentially offer more chances to hake individuals to attempt escape. Finally, for completeness and simplicity regarding onboard operations, an extension piece with no SMP was considered. The SMPs (single-braided 4mm polyamide in all cases) were inserted 1 m in front of the joint between the codend and the extension piece and were of different sizes depending on the gear design tested (Fig 1). The SMP_TS_ and SMP_BS_ were 2 m long, 1 m wide and had a mesh size of 82.70 mm ± 1.95 mm (mean ± SD). The SMP_TL_ was 2.81 m long, 1.70 m wide and had a mesh size of 80.00 ± 2.02 mm (mean ± SD). The SMP_BL_ was 3.56 m long, 1.90 m wide and had a mesh size of 77.30 ± 2.57 mm (mean ± SD).

Regarding the codend used in this fishery (70 mm mesh size, diamond mesh), it has been demonstrated that it retains undersized hake [19]. However, greatly increasing its mesh size to release small fish would potentially lead to escape of commercial sizes of hake and probably other fish species not considered in this study. Therefore, in this study a regular diamond mesh codend design (CD_D_) with slightly bigger mesh size that the commercial one was tested. The CD_D_ was 7.0 m long, double-braided 4 mm polysteel, forming 79.45 ± 2.01 mm (mean ± SD) meshes (Fig 1). The fishery, besides a variety of roundfish species, also includes important flatfish species. Since diamond mesh codends are typically more selective for flatfish than for roundfish species [48–50] a square mesh codend design (CD_S_) was also considered (Fig 1). The design guides made by Tokaç et al. [51] showed that a 60 mm mesh size with an opening angle of 90° would result on a *L*50 of approximately 25 cm for hake. However, in order to get a compromise between releasing undersized hake and not losing other important species for the fishery, the CD_S_ tested in this study was 7.0 m long, double-braided 3.5 mm polyethylene, forming 63.20 ± 1.73 mm (mean ± SD) meshes. The CD_S_ was constructed by turning the meshes in codend 45° (square meshes), and made of polyethylene twine, which is more deformable than polysteel [52] and facilitates fish escape (Fig 1).

Combining the five SMP designs with the two codend designs considered led to ten different gear combinations: (1) SMP_TS_ + CD_D_; (2) SMP_TS_ + CD_S_; (3) SMP_TL_ + CD_D_; (4) SMP_TL_ + CD_S_; (5) SMP_BS_ + CD_D_; (6) SMP_BS_ + CD_S_; (7) SMP_BL_ + CD_D_ (8) SMP_BL_ + CD_S_; (9) CD_D_; and (10) CD_S_ (Fig 1).

### Experimental design and sea trials

**Fig 2:**
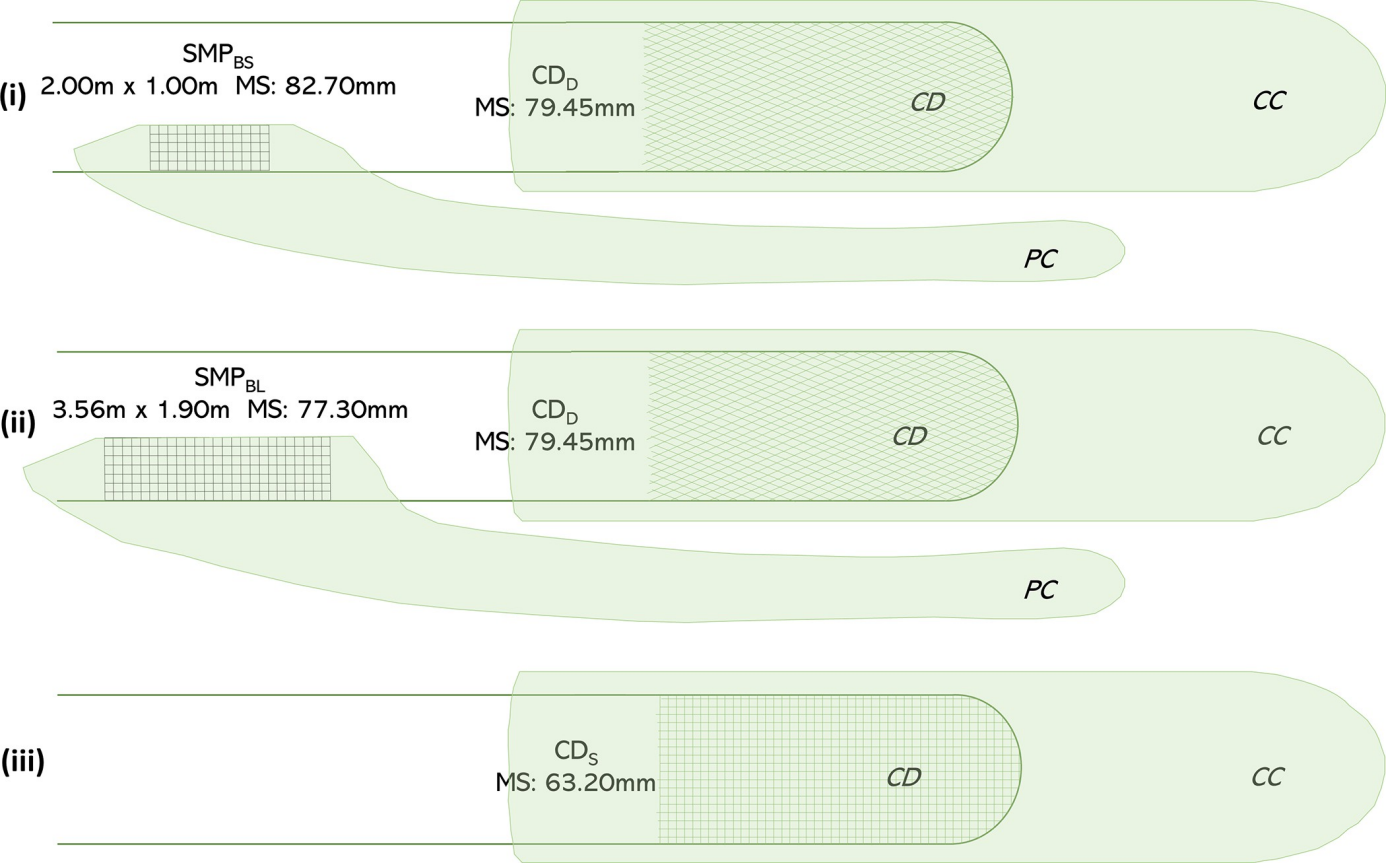
Experimentally tested gears with the covers used to collect fish. MS: mesh size.

Three gear designs were hence tested at sea: (i) SMP_BS_ + CD_D_; (ii) SMP_BL_ + CD_D_; and (iii) CD_S_ (Fig 2). From experimental designs (i) and (ii), selectivity data for the SMP_BS_, SMP_BL_ and CD_D_ were obtained, whereas selectivity data for the CD_S_ was obtained from design (iii). The selectivity data of SMP_TS_ and SMP_TL_ for hake and blue whiting was obtained from the sea trials conducted by Cuende et al. [23] and Cuende et al. [24], respectively. These two studies were carried out in 2017 and 2018 respectively, during same fishing period and similar fishing ground and depth. Using information from the experimental sea trials in Cuende et al. [23, 24], the selectivity of all ten gear combinations was subsequently modeled.

**Fig 3:**
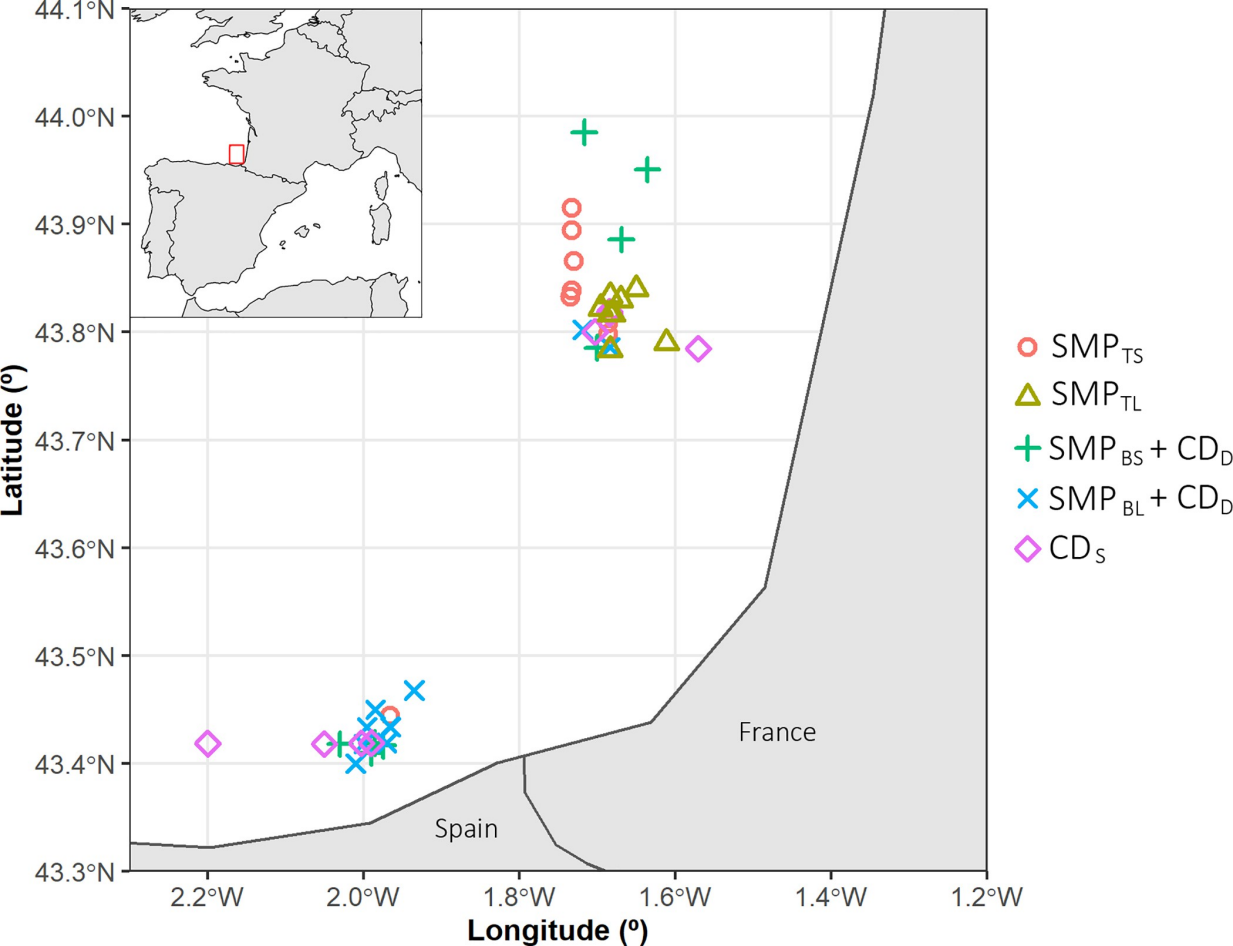
Sampling area and positions of the experimental hauls. Positions for SMP_TS_ and SMP_TL_ are also included [23, 24]. We used “ggplot2” [55] (under version 3.3.5) and “rnaturalearth” [56] (under version 0.1.0) within the R statistical environment [57] (R version 4.0.4) for mapping.

The sea trials in the current and previous studies [23, 24] included here were carried out on board the research vessel *Emma Bardan* (29 m length overall; 900 kW). The gear used in all experiments was a four-panel bottom trawl of the type GOC73 [53]. This trawl is built according to the standard bottom trawl survey manual for the Mediterranean [54]. The headline, sidelines, and fishing line were 35.7, 7.4, and 40.0 m long, respectively. The trawl was rigged with a set of Morgère doors (Morgère WH S8 type, 2.6 m^2^; 350 kg), 100 m sweeps, and a light rockhopper ground gear (with 3 × 40 kg chain + 15 kg chain on the bosom). While fishing, the trawl had a horizontal opening of approximately 16 m and a vertical opening between 2.7 and 3.2 m. Furthermore, all trials were carried out in the same period of the year (June) and in a similar area, within ICES divisions 8c and 8b, in Spanish and French waters (Fig 3).

Data was collected using the covered codend method [58]. For hauls where the gear design included SMPs, a dual-covered method was applied [59, 60]. In this case a cover was installed both over the SMP and the codend, and the data included the number of fish in the panel cover (*PC*), in the codend cover (*CC*) and in the codend (*CD*). In the gear configurations without SMP, a single cover was attached to the codend and the number of fish in the *CD* and *CC* were obtained (Fig 2). The same methodology was followed in Cuende et al. [23, 24] for SMP_TS_ and SMP_TL_.

The *PC* over the SMP_BS_ was 13.0 m long, with diamond meshes of 26.10 ± 0.91 mm (mean ± SD) (1.3 mm polyamide twine); the *PC* over the SMP_BL_ was 13.6 m long, with diamond meshes of 41.80 ± 0.85 mm (mean ± SD) (1.8 mm polyamide twine). Both were built based on the design by [61] and were equipped with nine floats (N-50/8 type; 135 mm diameter; 0.76 kg buoyancy each) on the top and leaded rope on the bottom to ensure expansion. The gear specifications for the SMP_TS_ and SMP_TL_ designs are available at Cuende et al. [23] and Cuende et al. [24], respectively. The *CC*s for CD_D_ and CD_S_ were 9 m long and made of 26.2 ± 0.41 mm (mean ± SD) (1.3 mm PA twine) and 33.70 ± 1.35 mm (mean ± SD) diamond meshes (3 mm polyamide twine), respectively. To ensure expansion of the covers and prevent obstruction of the codend meshes, nine pairs of floats (N-25/5 type; 100 mm diameter; 0.300 kg buoyancy each), eight kites (four per panel) and four chains (1 kg each) were respectively attached to the top, sides and bottom of the *CC*s. After each haul, the hake and blue whiting captured were measured to the nearest centimeter below. When the catch exceeded a maneuverable quantity in terms of the available time and crew for measuring the fish, randomly selected subsamples of the catch were taken, and the subsample ratio was calculated.

A flaw in the experimental design resulted in obvious differences in the mesh sizes used for SMP_BS_ and SMP_BL_ *PC*s and CD_D_ and CD_S_ *CC*s. Given that, we could not rule out that some of the smallest hake and blue whiting individuals might escape through the cover meshes. According to predictions made by Tokaç et al. [51], a 40 mm diamond mesh would be able to release hake between approximately 8 and 17 cm depending on mesh opening angle. Similarly, and based on the predictions for blue whiting in Cuende et al. [62], the same mesh size and mesh opening angles would release individuals ranging between 8 and 20 cm. Using data from length classes that could potentially escape through the covers could cause bias in the estimation of selection curves. For that reason, only individuals above 20 mm in the *PC* and above 15 mm length in the *CC* were considered in the analysis.

#### Models used for SMP and codend size selection estimation

The selectivity models applied were specific for the SMPs and codends independently, meaning that even though some hauls included data for SMP and codend size selection, the data collected for each gear compartment was analyzed separately. Regarding the diamond mesh codend, since the codend used together with SMP_BS_ and SMP_BL_ was the same, the codend data for those hauls was analyzed together, resulting in a larger and more robust dataset.

To estimate the retention probability (*r*_*SMP*_(*l*)) for SMP_BS_ and SMP_BL_, the fraction of fish escaping through the SMP was compared to the fraction that did not escape through it. Assuming that the fate of each fish is independent of that of other fish, the number of individuals of a specific length class *l* present in the *PC* (*nPC*) was compared to the sum of the individuals present in the *CD* and *CC* (*nCD* + *nCC*). The experimental data in the analysis was thus treated as two-compartment data and described using a binomial distribution with length-dependent probabilities of being retained by the SMP *r*_*SMP*_(*l*).

A fish entering SMP zone can be size-selected if it contacts the panel and its body size, shape, and orientation allows it to pass through the meshes. For the fish that contact the SMP we therefore assume that the length-dependent retention probability can be sufficiently well modeled by a *logit* function [58], defined by the parameters *L*50_*SMP*_ (length at which a fish contacting the panel has a 50% chance of escaping through the SMP) and *SR*_*SMP*_ (difference between the lengths at which a fish contacting the panel has 75% and 25% chances of escaping through the SMP). However, because some fish may not come into contact with the SMP, the size selection process was modeled based on a *CLogit* size selection model [63], which has shown to be sufficiently flexible to describe the process [22, 64]. The *CLogit* model estimates the available size selection for the SMP through the parameter *C*_*SMP*_, which quantifies the probability that a fish entering the SMP zone will contact the SMP and be subject to a size-dependent probability of escaping through it (selectivity contact). We assumed that the likelihood of *C*_*SMP*_ can be modeled by a single length independent number that ranges between 0.0 and 1.0. If *C*_*SMP*_ is equal to 1.0, all fish contact the SMP, whereas if *C*_*SMP*_ is equal to 0.0, none do. Therefore, the length-dependent SMP retention probability, *r*_*SMP*_(*l*), can be modeled by:

$$r_{SMP}(l,C_{SMP},{L50}_{SMP},{SR}_{SMP})=CLogit(l,C_{SMP},{L50}_{SMP},{SR}_{SMP})=1.0-C_{SMP}+C_{SMP}\times logit(l,{L50}_{SMP},{SR}_{SMP})$$ (1)

where

$$logit(l,L50,SR)=\frac{exp(ln(9)\times (l-L50)/SR)}{1.0+exp(ln(9)\times (l-L50)/SR)}$$ (2)

For codend retention probability (*r*_*CD*_(*l*)), the fraction of fish in the *CD* was compared to the fraction of fish in the *CC*. We assumed that the retention likelihood could be modeled using a binomial distribution with length-dependent probabilities for being retained in the codend (*r*_*CD*_(*l*)) by a *logit* model with parameters *L*50_*CD*_ and *SR*_*CD*_:

$$r_{CD}(l,{L50}_{CD},{SR}_{CD})=Logit(l,{L50}_{CD},{SR}_{CD})$$ (3)

#### Estimation of SMP and codend size selection

The parameters *C*_*SMP*_, *L*50_*SMP*_, *SR*_*SMP*_, *L*50_*CD*_, and *SR*_*CD*_ were estimated simultaneously on a haul-by-haul basis. We used a maximum likelihood estimation (MLE) method, pooling the experimental data over the hauls *j* (*1* to *m*) for each specific gear and minimizing the following expression [23, 24]:

$$-\sum_{l}\sum_{j=1}^{m}\left\{(\frac{nCD_{lj}}{qCD_{j}}+\frac{nCC_{lj}}{qCC_{j}})\times \ln\left(r_{SMP}(l,C_{SMP},{L50}_{SMP},{SR}_{SMP})\right)+\frac{nPC_{lj}}{qPC_{j}}\times \ln\left(1.0-r_{SMP}(l,C_{SMP},{L50}_{SMP},{SR}_{SMP})\right)\right\}$$ (4)

whereas codend size selectivity was estimated by:

$$-\sum_{l}\sum_{j=1}^{m}\left\{\frac{nCD_{lj}}{qCD_{j}}\times \ln\left(r_{CD}(l,{L50}_{CD},{SR}_{CD})\right)+\frac{nCC_{lj}}{qCC_{j}}\times \ln\left(1.0-r_{CD}(l,{L50}_{CD},{SR}_{CD})\right)\right\}$$ (5)

For each haul *j* and length class *l*, *nCD*_*lj*_, *nPC*_*lj*_, *and nCC*_*lj*_ are the numbers of individuals length-measured in the *CD*, *PC*, and *CC*, respectively; and *qCD*_*j*_, *qPC*_*j*_, and *qCC*_*j*_ are their respective subsampling factors (ratio of length-measured to total number of fish in each compartment). The summation is over the length classes (each 1 cm wide).

The models were validated based on p-value estimations and model deviance versus degrees of freedom [58]. If the p-value was < 0.05 and deviance was much greater than the degrees of freedom, the residuals were inspected to determine whether the discrepancy between model and experimental data was the result of over-dispersion. On the other hand, a p-value > 0.05 means that it cannot be ruled out that the difference observed between the model and the data is coincidental.

The confidence intervals (CIs) for the average size selection were estimated using a double bootstrap method. This approach is identical to the one described in Millar and Fryer [65] and Herrmann et al. [66], and takes both within-haul and between-haul variation into consideration. Each of the 1,000 bootstrap repetitions conducted resulted in a ‘pooled’ set of data used to estimate the Efron percentile [67] 95% CIs for the selection curve and its parameters [68]. We applied the software tool SELNET [68] for the size selection analysis and used the double bootstrap method implemented in this tool to obtain CIs for the size selection curve and the corresponding parameters.

### Size selection models for combined SMP and codend designs

To estimate the retention probability of the different gear combinations (SMP_TS_ + CD_D_; SMP_TS_ + CD_S_; SMP_TL_ + CD_D_; SMP_TL_ + CD_S_; SMP_BS_ + CD_D_; SMP_BS_ + CD_S_; SMP_BL_ + CD_D_; SMP_BL_ + CD_S_; CD_D_ and CD_S_), a sequential combination of the different SMP and codend designs was modeled. The combined retention probability of the specific gear combination (*r*_*comb*_(*l*)) was modeled using the following generic model [22, 23, 60]:

$$r_{comb}(l,C_{SMP},{L50}_{SMP},{SR}_{SMP},{L50}_{CD},{SR}_{CD})=r_{SMP}(l,C_{SMP},{L50}_{SMP},{SR}_{SMP})\times r_{CD}(l,{L50}_{CD},{SR}_{CD})$$ (6)

Due to the differences in minimum fish length included in the analyses (20 mm for *PC* and 15 mm for *CC*), the selectivity in the different compartments was analyzed separately. Therefore, although SMP_BS_ + CD_D_ and SMP_BL_ + CD_D_ combinations were experimentally tested, their combined retention was also modeled by Eq (6).

### Comparison between different gear designs

To investigate whether and how the different gear designs perform with respect to each other, we quantified (a) changes in absolute selectivity, by using the delta selectivity [69]; (b) catch profile, by estimating the structure of the population caught; and (c) potential consequences for the fishery, using exploitation pattern indicators [70].

#### Treatment trees

**Fig 4:**
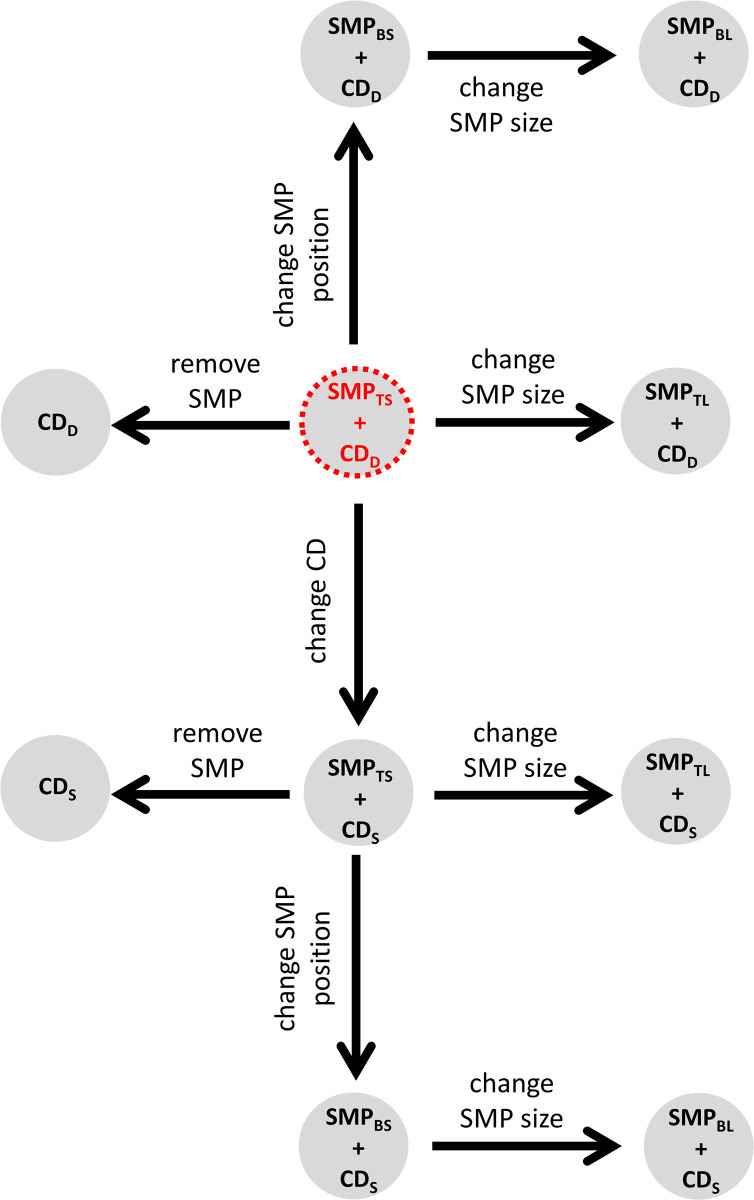
Treatment tree diagram. Arrows represent the delta comparisons carried out. Red circle indicates the reference gear design.

To investigate the effect of the gear modifications implemented on size selectivity and catch profile of hake and blue whiting, treatment trees were used. Delta selectivity was estimated by subtracting the predicted, species-specific, absolute selectivity of two gear designs to identify size ranges where there was a significant change in selectivity [69]. The pooled delta selectivity for each gear combination were arranged in a tree-like structure, starting with a reference gear design, which was connected stepwise to the remaining gear designs. The reference gear design established was the one used by the fleet today, SMP_TS_ + CD_D_. Every step forward changed to a gear design (treatment gear design) where a unique modification was implemented (Fig 4). That modification could be increasing SMP size, changing SMP position, removing SMP or changing codend mesh geometry (Fig 4).

In each step (Fig 4), the delta selectivity curves and size selectivity for the treatment gear, baseline gear and reference gear designs were shown with the corresponding CIs. Delta selectivity curves showed the difference in the retention probability between a gear design with an implemented modification (treatment gear) and its baseline gear design. To infer the difference in retention probability, the following generic delta curve (*Δr*(*l*)) was applied:

$$\Delta r(l)=r_{treatment}(l)–r_{baseline}(l)$$ (7)

where *r*_*treatment*_(*l*) is the retention probability value of a specific gear which has implemented a modification on its design, and *r*_*baseline*_(*l*) is the retention probability value of the baseline gear design in each pairwise comparison.

Efron 95% CIs for *Δr*(*l*) were obtained based on the two bootstrap populations of results (1,000 bootstrap repetitions in each) for both *r*_*treatment*_(*l*) and *r*_*baseline*_(*l*). As the bootstrap resampling was random and independent for the two groups of results, it is valid to generate the bootstrap population of results for the difference based on (7) using the two generated bootstrap files [66]:

$${\Delta r(l)}_{i}={r_{treatment}(l)}_{i}-{r_{baseline}(l)}_{i}\;i\in [1\ldots 1000],$$ (8)

where *i* denotes the bootstrap repetition index. Significant differences in size selection between gears were obtained if the 95% CIs for the delta curves had length classes that did not overlap 0.0.

Following the same approach, a treatment tree was applied to depict catch profiles of the treatment and the reference gear designs. Each step in the tree showed differences in the fish population retained by the treatment gear compared to the reference gear design (Fig 4). To estimate the differences in the fish population retained, the size selection curves predicted for each design combination were applied to the population of hake and blue whiting entering the fishing gear by:

$${nr}_{l}={nPop}_{l}\times r_{comb}(l)$$ (9)

Efron 95% CIs for the average populations retained were estimated using a double bootstrap method. The population applied throughout this process was the population entering the gear during the experimental fishing with SMP_BS_ + CD_D_. In this gear design, *PC* and *CC* mesh sizes were suitable for retaining the whole length class ranges of hake and blue whiting and the individuals fished covered a wide range of sizes. Therefore, SMP_BS_ + CD_D_ was assumed to be representative of the population fished during the trials with the remaining gear designs. These treatment trees show the mean population retained of the treatment gear (with CIs) and the population retained by the reference gear design.

#### Exploitation pattern indicators

To investigate how applying the different design combinations considered would affect the catch pattern in the fishery, we estimated the value of three exploitation pattern indicators, *nP*^*−*^, *nP*^*+*^ and *nDiscard*, for each gear design. These indicators are often used in fishing gear size selectivity studies to supplement assessment solely based on selectivity curves [22, 25, 38, 71–73]. Specifically, the percentage of individuals retained below (*nP*^*−*^) and above (*nP*^*+*^) the species-specific minimum conservation reference size (MRCS) was estimated, as well as the discard ratio (*nDiscard*), which quantifies the fraction of hake and blue whiting below MCRS in the total catch (in %). MCRS for hake is 27 cm. For blue whiting, which does not have an MCRS, we used its estimated marketable size limit, 18 cm length. That length is based on a regulation that establishes a maximum of 30 individuals of blue whiting per kilo for commercialization [74, 75].

Since these indicators are affected by the populations fished, which may vary depending on factors such as fishing period and area, we analyzed the catch patterns of the different design combinations considered for different population scenarios. The different populations corresponded to selectivity data obtained in different fishing areas in the Bay of Biscay (ICES 8abd) and Western Iberian waters (ICES 9a) in different years (in between 2011 and 2019). The exploitation indicators calculated for those scenarios were used to discuss the most promising gear design for the fishery under study.

The indicators were estimated for the ten combined gear designs considered by:

$$\begin{array}{l} {nP}^{-}=100\times \frac{\sum_{l<MCRS}\left\{r_{comb}(l)\times {nPop}_{l}\right\}}{\sum_{l<MCRS}\left\{{nPop}_{l}\right\}},\\ {nP}^{+}=100\times \frac{\sum_{l>MCRS}\left\{r_{comb}(l)\times {nPop}_{l}\right\}}{\sum_{l>MCRS}\left\{{nPop}_{l}\right\}},\\ nDiscard=100\times \frac{\sum_{l<MCRS}\left\{r_{comb}(l)\times {nPop}_{l}\right\}}{\sum_{l}\left\{r_{comb}(l)\times {nPop}_{l}\right\}} \end{array}$$ (10)

Indicators *nP*^*−*^, *nP*^*+*^ and *nDiscard* were estimated with uncertainties for each species and population scenario, using the bootstrap set for *r*_*comb*_(*l*) and *nPop*_*l*_, specifically, by first calculating the values for the indicators based on the result of each bootstrap repetition for *r*_*comb*_(*l*) and *nPop*_*l*_ in (10) to obtain a bootstrap set for the indicator values. Efron 95% CIs were estimated for each of the indicators based on the resulting bootstrap set.

To visualize and categorize multiple exploitation pattern indicator results, a traffic light system procedure was implemented, using red, yellow and green colors. Specifically, the colors express indicator values regarding how ‘favorable’ or ‘poor’ they are with respect to regulations. In simple terms, data in green color represents satisfactory/safe outcomes, while data in red represents dangerous outcomes. The conditions in-between are transitional outcomes represented in yellow. The change in colors is gradual, from green to yellow and from yellow to red depending on the value of the indicator. For example, an ideal fishery where *nP*^*−*^ and *nDiscard* are low (close to 0) and *nP*^*+*^ is high (close to 100) would be represented by a green color, intermediate values would shift to a yellow/orange color, while very high *nP*^*−*^ and *nDiscard* or low *nP*^*+*^ would be indicated by red.

## Results

### Overview of sea trials

During the sea trials, selectivity data for SMP_BS_, SMP_BL_, CD_D_ and CD_S_ was obtained for hake and blue whiting from a total of 33 and 32 experimental hauls, respectively. Specifically, eight experimental hauls with SMP_BS_ were carried out, nine with SMP_BL_, seventeen with CD_D_ and eight with CD_S_ were carried out for hake. For blue whiting, eight experimental hauls with SMP_BS_, nine with SMP_BL_, sixteen with CD_D_ and seven with CD_S_ were carried out. The towing speed was between 2.9 and 3.0 knots, and towing depths varied between 99 and 126 m. The two covers enabled separate collection and measurement of the individuals retained by the *CD*, *CC*, and *PC* per haul and length class. Length-measured individuals included 11,665 hake and 10,463 blue whiting. In general, the models used seemed to explain the experimental data adequately, which was confirmed by the fit statistics (p-value > 0.05) (Table 1). The poor p-value associated to SMP_TS_ for blue whiting was probably due to overdispersion in the data created by heavy subsampling ratios [23], as the experimental data and the fitted escape probability curve showed no clear deviation patterns.

**Table 1:** Selectivity parameters for hake and blue whiting for the different SMP and codend designs considered in the study.

	SMP_TS_	SMP_TL_	SMP_BS_	SMP_BL_	CD_D_	CD_S_
Hake						
*L*50	37.07 (21.22–37.10)	32.07 (31.04–32.10)	35.03 (0.10–35.09)	31.33 (15.02–34.73)	15.68 (12.47–17.51)	23.49 (22.82–24.46)
*SR*	0.10 (0.10–7.42)	0.10 (0.10–0.10)	0.10 (0.10–27.08)	6.51 (0.10–19.72)	7.92 (5.07–11.84)	4.36 (3.73–4.92)
*C*_*SMP*_	0.01 (0.00–0.02)	0.02 (0.01–0.03)	0.05 (0.02–1.00)	0.38 (0.27–1.00)	-	-
p-Value	0.972	0.05	0.8735	0.6995	0.4420	0.8543
Dev	59.29	102.44	14.73	23.66	36.57	25.46
DOF	82	81	22	28	36	34
Blue whiting						
*L*50	27.62 (23.14–34.76)	32.39 (29.81–197.57)	28.97 (0.10–56.51)	0.10 (0.10–1.00)	22.88 (20.76–24.12)	27.06 (26.70–27.44)
*SR*	8.99 (0.10–15.73)	1.99 (0.10–48.72)	0.10 (0.10–4.60)	27.73 (0.10–40.50)	4.37 (3.60–5.61)	3.32 (2.85–3.84)
*C*_*SMP*_	0.27 (0.21–0.38)	0.45 (0.26–0.66)	0.00 (0.00–1.00)	0.18 (0.01–1.00)	-	-
p-Value	<0.001	0.31	0.9793	0.6226	0.1159	0.3349
Dev	105.10	25.81	4.21	8.99	21.70	19.96
DOF	40	23	12	11	15	18

Selectivity parameters estimated, 95% CIs (in brackets) and fit statistics are shown. Selectivity parameters and fit statistics from trials in Cuende et al. [23, 24] are also shown.

The number of hauls, individuals measured and haul characterization for experimental trials including SMP_TS_ and SMP_TL_ designs are available in Cuende et al. [23, 24]. Selectivity parameters and fit statistics for these gear designs are also included in Table 1.

### Size selectivity of individual SMP and codend designs

The selectivity parameters shown in Table 1 demonstrate that the contact probability resulted on the highest values for hake when the SMP_BL_ was used. It was estimated that 38% (CI: 27%–100%) of the hake contact the SMP_BL_ while not more than 5% contact the remaining SMP designs. The contact probability values for blue whiting were highest when the SMP designs were located at the top panel, being of 45% (CI: 26%–66%) when SMP_TL_ was used and of 27% (CI: 21%–38%) with SMP_TS_. Regarding codend selectivity, CD_S_ released smaller individuals than CD_D_, since CD_S_ design showed a higher *L*50 value and lower *SR* value than CD_D_ for both species.

**Fig 5:**
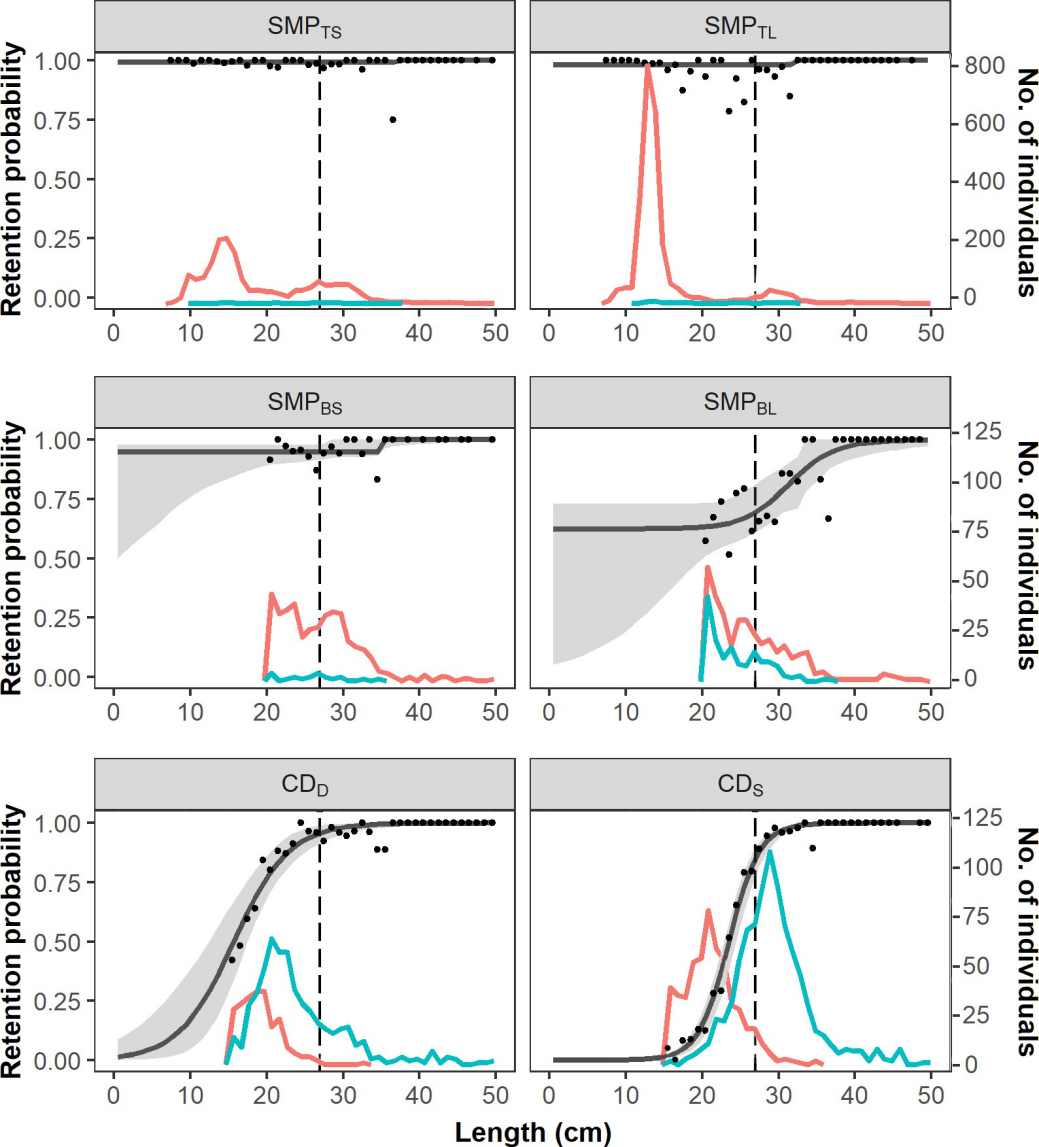
Length-dependent retention probabilities for hake. Retention probability curves (black line) with corresponding CIs (grey bands) and experimental rates (black dots) for the different SMP and codend configurations for hake. Vertical dashed lines show the MCRS for hake: 27 cm. The number of individuals escaped (red lines) and retained (blue lines) by each design are also shown.

**Fig 6:**
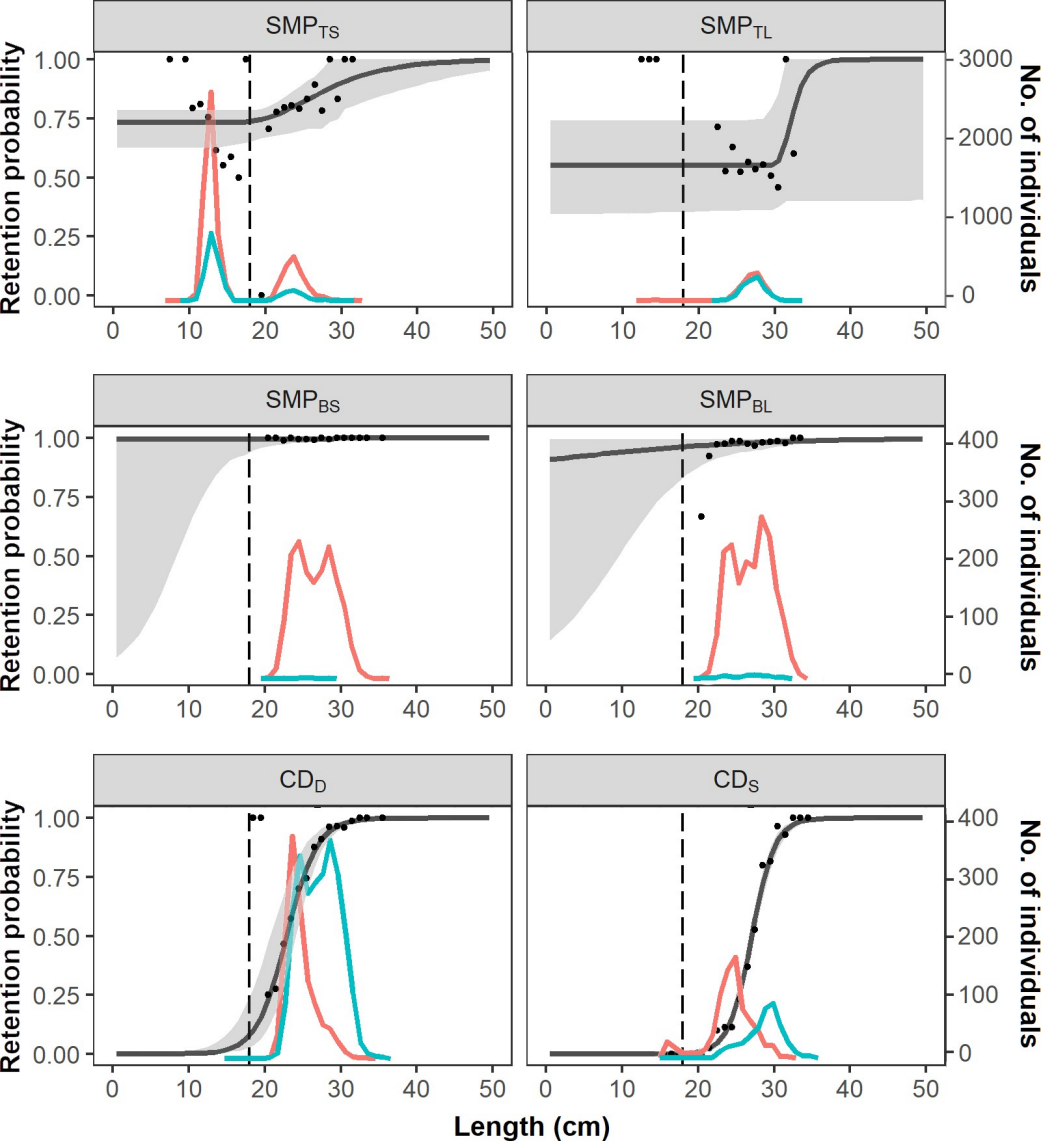
Length-dependent retention probabilities for blue whiting. Retention probability curves (black line) with corresponding CIs (grey bands) and experimental rates (black dots) for the different SMP and codend configurations for blue whiting. Vertical dashed lines show the minimum marketable size for blue whiting: 18 cm. The number of individuals escaped (red lines) and retained (blue lines) by each gear are also shown.

The size selection curves for hake show a flat shape for the designs consisting of an SMP at the top panel (SMP_TS_ and SMP_TL_), and changes in the pattern occur when the SMP’s position is moved to the bottom panel, especially when the SMP_BL_ is used (Fig 5). The patterns observed are the opposite for blue whiting, as the SMP designs at the bottom panel (SMP_BS_ and SMP_BL_) do not have any effect on its escape probability, whereas when it is placed at the top panel, the retention probability of smaller length classes is reduced to 73.26% and 55.08% for SMP_TS_ and SMP_TL_, respectively (Fig 6).

Regarding codend size selectivity, CD_S_ increases the size at which the codend starts retaining fish with respect to CD_D_ for both hake and blue whiting (Figs 5 and 6). For example, *L*25 of CD_D_ for hake is 12 cm and increases to 21 cm for CD_S_; similarly, it increases from 21 cm to 25 cm for blue whiting.

### Treatment trees

**Fig 7:**
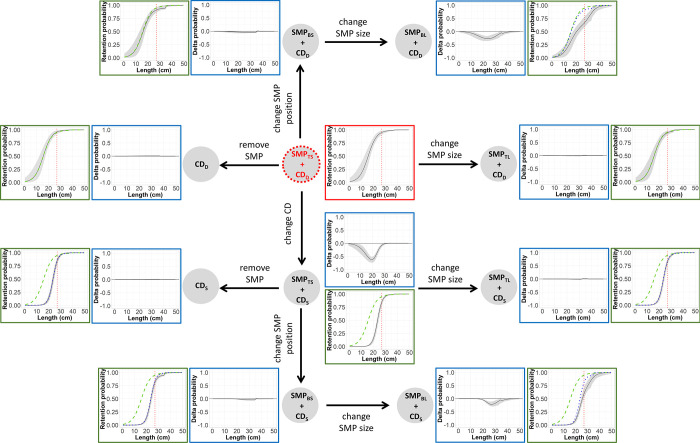
Size selection treatment tree for hake. Delta comparisons (blue boxes) carried out (represented by arrows), which include delta curves for each modification applied in the gear (black line) with its corresponding CIs (grey bands), are shown. Each step also includes selectivity plots (green boxes) showing: selection curves for the treatment gear design (black line) with CIs, baseline gear design (blue dots) and reference gear design (green dashed lines). Vertical red dotted lines correspond to the MCRS: 27 cm.

The treatment tree for hake shows that regardless of codend design, changing the SMP position from the top panel to the bottom panel as well as increasing its size significantly decreases the retention probability compared to the reference gear design (SMP_TS_ + CD_D_). However, changing codend geometry from CD_D_ to CD_S_ has a greater effect on the gear’s retention probability by decreasing it to a maximum of 61.97% (CI: 51.76–73.70%) for hake of 20 cm (Fig 7). The size selection curves in the treatment tree reveal that all gear designs including the CD_S_ release more undersized hake, especially when combined with the SMP_BL_. They also show that the retention probability curves for the gear designs with CD_D_ are less steep than when combined with CD_S_ (have higher *SR*), which result in a lower retention probability for hake. CIs for gears combined with CD_S_ are narrower than for those with CD_D_ (Fig 7).

**Fig 8:**
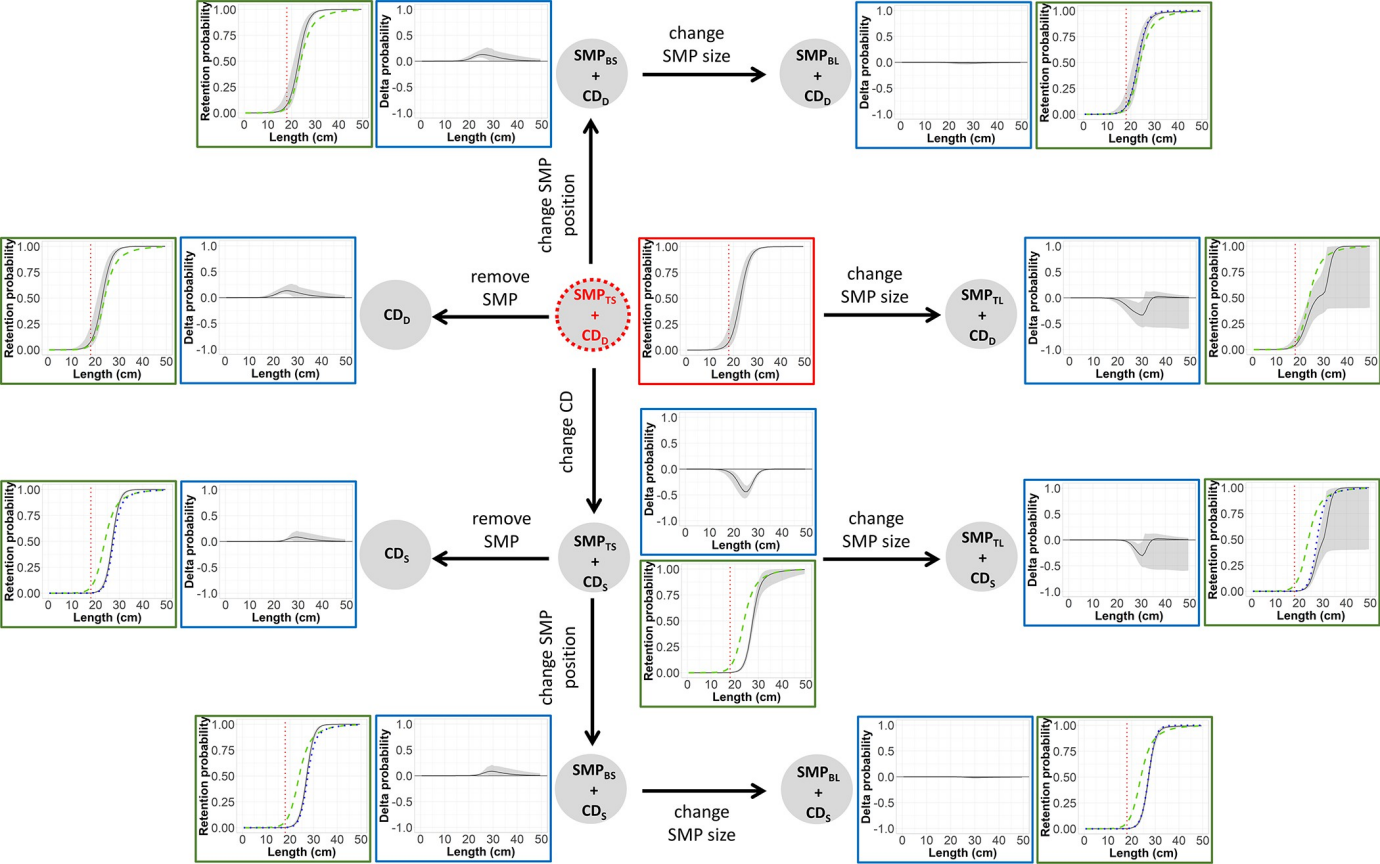
Size selection treatment tree for blue whiting. Delta comparisons (blue boxes) carried out (represented by arrows), which include delta curves for each modification applied in the gear (black line) with corresponding CIs (grey bands), are shown. Each step also includes selectivity plots (green boxes) showing: size selection curves for the treatment gear design (black line) with CIs, baseline gear design (blue dots) and reference gear design (green dashed lines). Vertical red dotted lines correspond to the minimum marketable size: 18 cm.

Regarding blue whiting, either removing the SMP or changing its position to the bottom panel would retain significantly more individuals above their marketable size. For example, using CD_D_ and CD_S_ without any SMP can retain up to 13.10% (CI: 5.98%–25.72%) and 8.86% (CI: 0.00%–21.03%) more individuals of 26 and 29 cm, respectively (Fig 8). Similarly, in gears composed by CD_D_ and CD_S,_ changing the position of the SMP from SMP_TS_ to SMP_BS_ retain up to 13.06% (CI: 6.71%–23.44%) and 8.76% (CI: 0.00%–21.02%) more individuals of 25 and 29 cm, respectively. Conversely, increasing the size of the SMP increases the escape of commercial-size individuals. Also, SMP_TS_ + CD_S_ would significantly affect the retention probability of blue whiting by releasing up to 43.75% (CI: 30.52%–57.09%) more individuals of 24 cm than the SMP_TS_ + CD_D_ gear design. Additionally, the size selection curves show that all gear designs considered would mostly fish individuals above the respective marketable size since the retention probability of individuals below 18 cm is lower than 6% of the total catch in every case. Designs with CD_S_ release 100% of individuals below 18 cm and achieve high retention probabilities (above 50%) for fish >25 cm (Fig 8).

**Fig 9:**
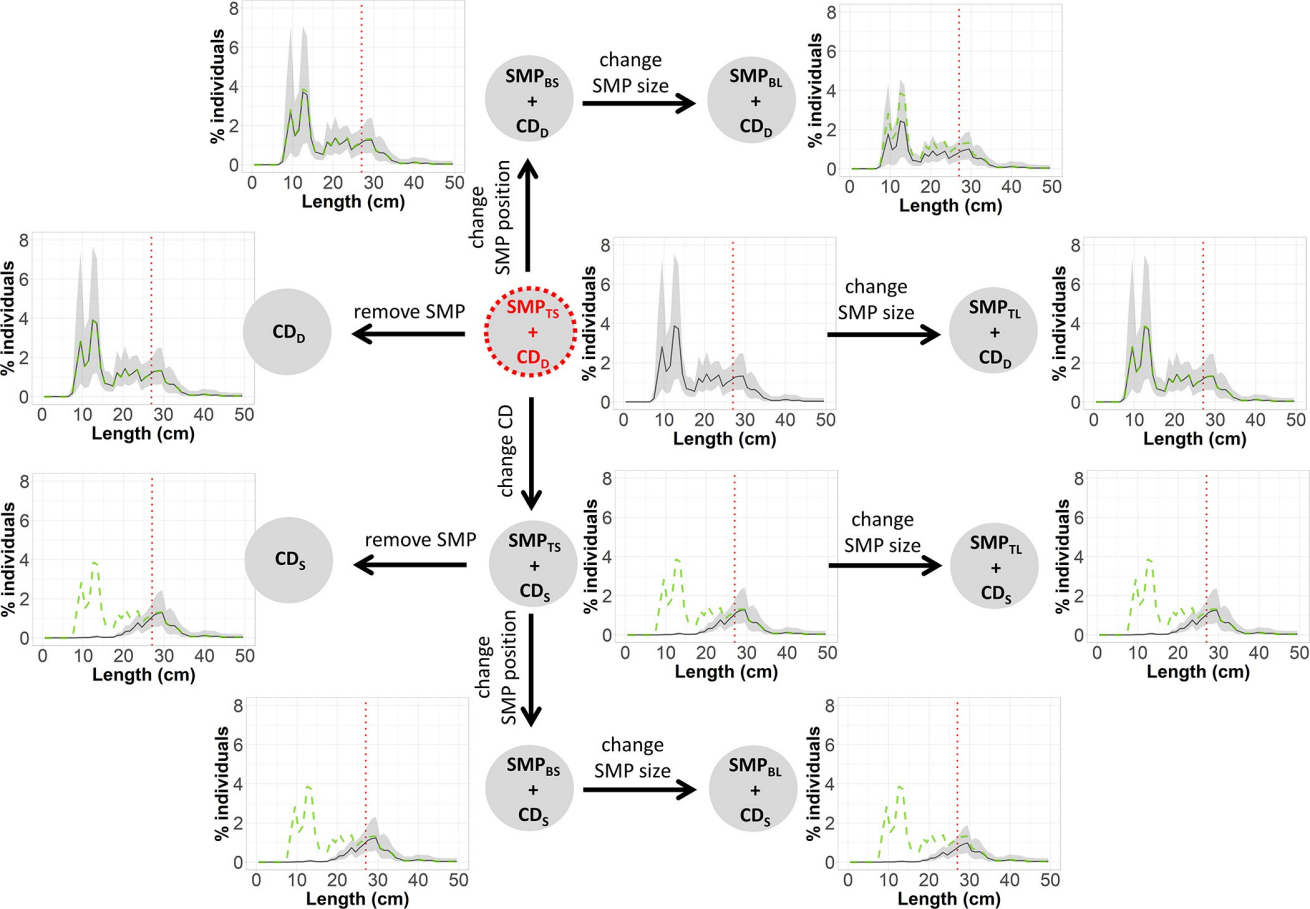
Treatment tree of the population structure fished for hake with the different gear designs. Includes the fished population structure (black line) for each gear design and CIs (grey bands) and the population structure fished by the reference gear design (SMP_TS_ + CD_D_) (green dashed lines). Vertical red dotted lines correspond to the MCRS: 27 cm.

**Fig 10:**
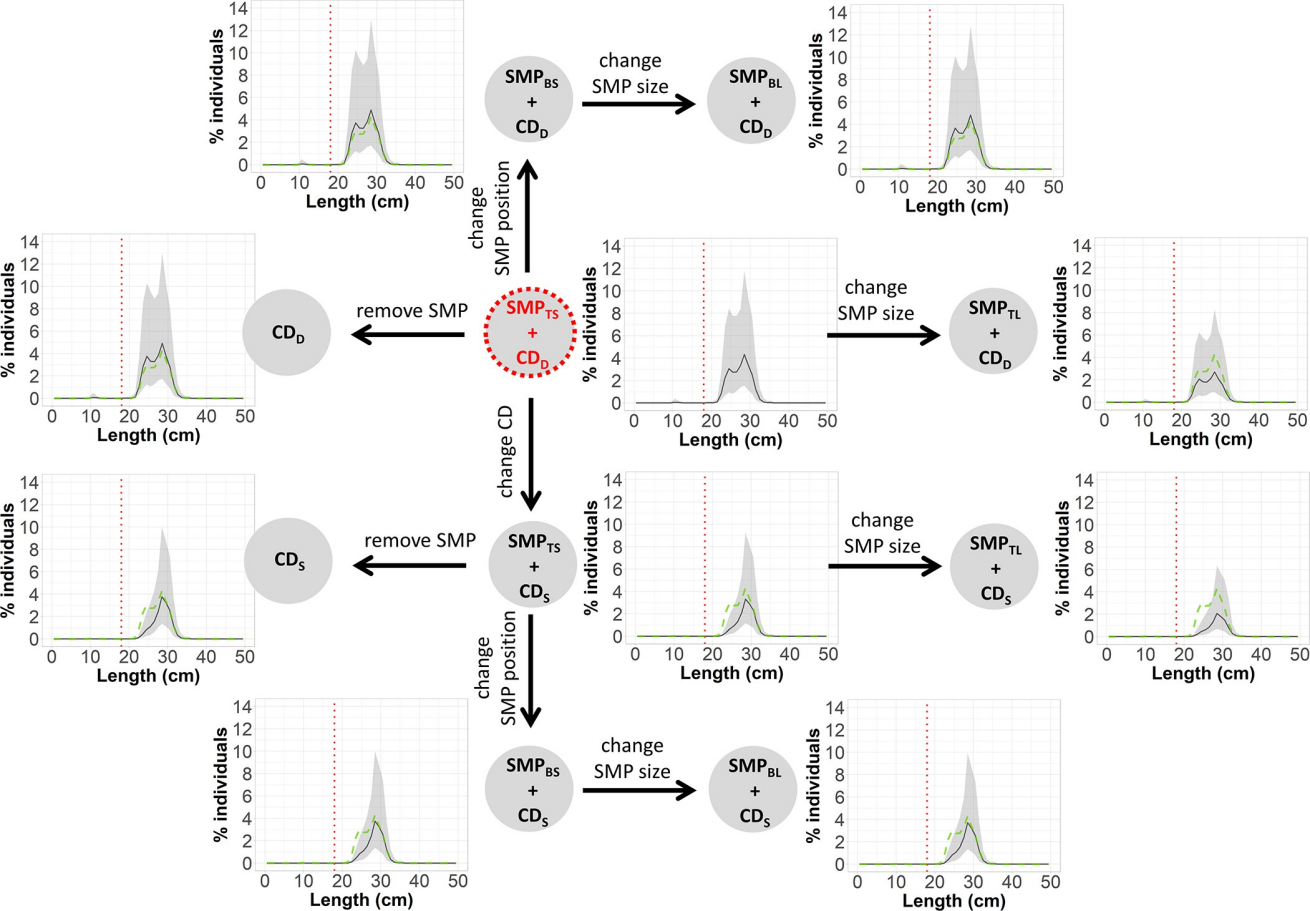
Treatment tree of the population structure fished for blue whiting with the different gear designs. Includes the fished population structure (black line) for each gear design and CIs (grey bands) and the population structure fished by the reference gear design (SMP_TS_ + CD_D_) (green dashed lines). Vertical red dotted lines correspond to the minimum marketable size: 18 cm.

The catch profiles showed that the proportion of catch composed of undersized individuals (i.e. < MCRS) can vary significantly when using the different gear designs (Figs 9 and 10). For hake, the designs with CD_S_ catch larger individuals, while CD_D_, even though some SMP designs (like SMP_BL_) can release a higher proportion of undersized fish, mostly retains undersized fish (Fig 9). For blue whiting the plots show that the catch pattern of every gear design is composed by individuals above their minimum marketable size. In this case, those gear combinations with CD_D_ retain higher proportion of fish above MCRS that the gears combined with CD_D_ (Fig 10).

### Exploitation pattern indicators

**Fig 11:**
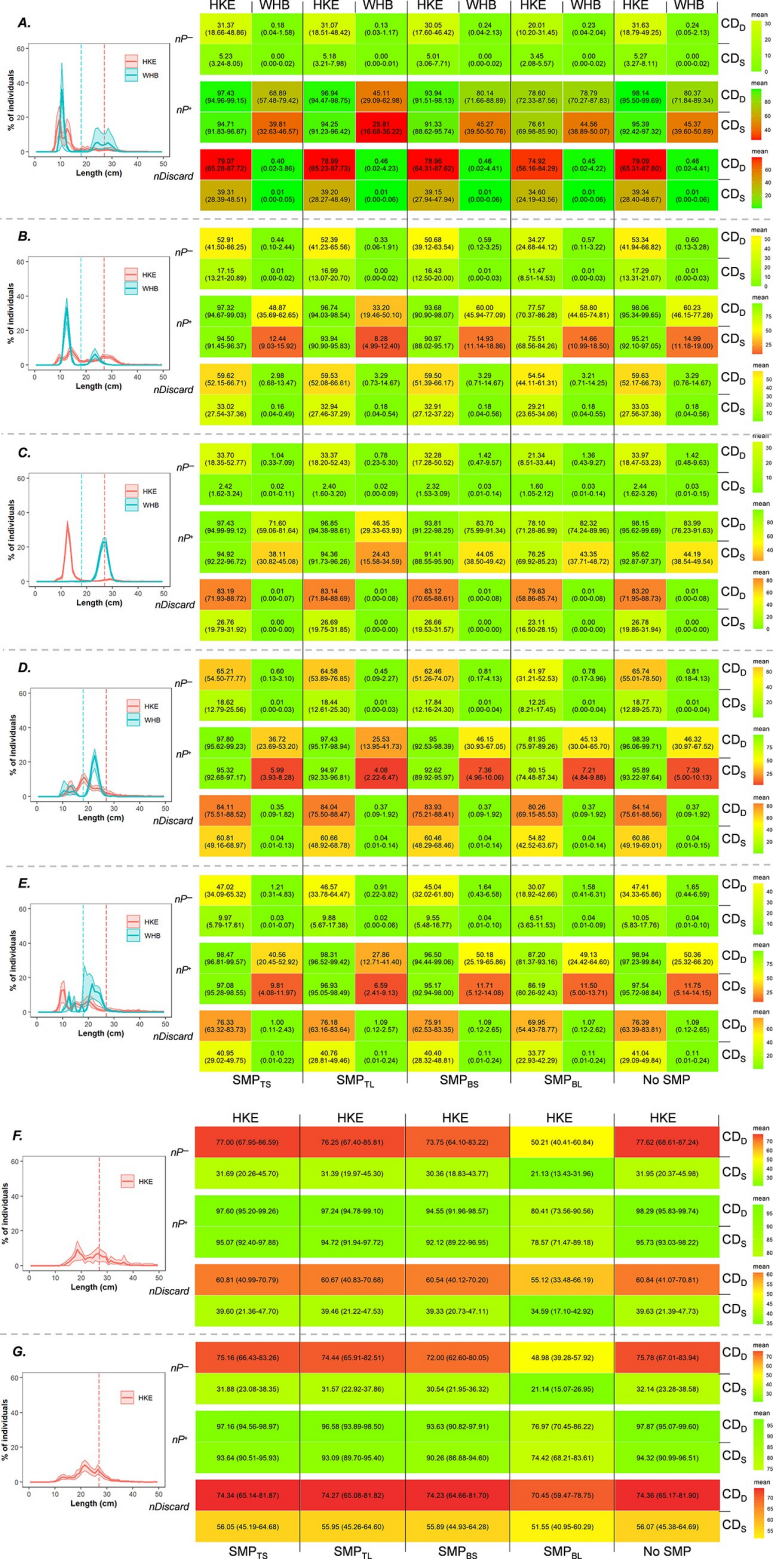
Diagram of the exploitation pattern indicators for every gear combination and species following a traffic light system. A to G rows show different fish populations. In the left side, population structures for hake (HKE) and blue whiting (WHB) are shown, with vertical dashed lines representing the MCRS of hake (27 cm) and the estimated minimum marketable size of blue whiting (18 cm). In the right side, the traffic light diagrams show the indicators values (%), with green indicating ‘satisfactory’ and red ‘unsatisfactory’.

To identify the most favorable design regarding catch patterns, the indicators *nP*^*−*^, *nP*^*+*^ were estimated for all gear designs and population scenarios considered. The highest proportion of undersized hake was always retained when the population structure largely comprised individuals close to the MCRS. For example, for those populations composed mainly of hake below 20 cm (Fig 11*A*, 11*C*), *nP*^*−*^ and *nP*^*+*^ show greenish colors for almost all designs, meaning that they have a low probability of retaining them. However, when the population includes individuals closer to the MCRS but still below 27 cm, the retention of sized individuals remains high while yellow-red colors are expressed for undersized individuals in most of the gear designs (Fig 11*B*, 11*D–*11*G*). In general, although the catch of individuals above MCRS is higher when any SMP design is used together with CD_D_, *nDiscard* shows lower values when these are combined with CD_S_. For blue whiting, the results show mostly yellow-red colors for the capture of individuals above 18 cm, meaning low efficiency in retaining these individuals. When the blue whiting population is composed of individuals above the respective marketable size but larger than 22 cm (Fig 11*C*), the indicators *nP*^*+*^ and *nDiscard* show better values for all gear designs, especially for SMP_BS_ + CD_D_ and CD_S_.

When the exploitation of both species is considered together, the gear designs with fewer undersized retention are those combined with CD_S_. However, CD_S_ also has a higher release of individuals above MCRS, which could make the fishing activity less efficient. These results show that the exploitation pattern of hake and blue whiting can be greatly influenced by making small changes in the gear design.

## Discussion

Adapting the selectivity of fishing gears is an important strategy to achieve desired catch patterns and meet management objectives. The diversity of unwanted species and sizes caught in fisheries has led to the development of a vast array of gear designs and consequently to a great deal of literature focusing on the effect of those designs on size and species selectivity [4, 76, 77]. The approach used in this study makes best use of existing knowledge on size selectivity in the Basque bottom trawl fishery and leads to new insights about the potential for its improvement. This approach allowed us to quickly inspect a number of potential gear modifications based on few experimental trawl designs and data already available. Specifically, the gear combinations implemented led to the identification of ten potentially applicable gear designs that could help the fishery meeting the management requirements (e.g. European Landing Obligation [78]).

The effect of multiple gear modifications on the size selectivity and catch patterns of hake and blue whiting was systematically illustrated using treatment trees. This tool presented all the gear designs by graphically illustrating predicted retention probabilities, delta estimates among designs and catch profiles of different population scenarios, and provided detailed information about the contribution of the different components of the gear to its overall performance.

Treatment trees for hake demonstrated that changing the position of the panel to the lower netting, along with increasing its size (SMP_BL_), decreased the retention probability of undersized individuals. These results are in line with those of Cuende et al. [24], who showed that panel position could be a key factor to improve the release of undesired and non-target hake, since positioning the SMP in the lower panel could favor the escape of species that swim closer to the lower panel of the trawl [23]. In contrast, the 60 mm mesh size located in the lower panel used by Nikolic et al. [47] in the Bay of Biscay’s *Nephrops* fishery did not show to have any effect on hake catches. However, their study was based on data that included total catches and mean lengths, and the population fished was unknown. Therefore, we believe that our results cannot be directly compared to those. The results in this study showed that if only the position (SMP_BS_) or size (SMP_TL_) of the SMP was changed, the contact probability between the fish and the SMP was not improved compared to the SMP_TS_ design. However, the escape probability comparison between SMP_BL_ and SMP_TL_ should be interpreted carefully because SMP_BL_ had a bigger dimension than SMP_TL_. The results demonstrate that increasing hake chances to contact the different SMPs (placed at the top and the bottom panel, respectively) is only significantly effective when placed at the bottom.

Regarding codend mesh geometry, CD_S_ significantly increased the escape probability of hake, and this increased even more when combined with SMP_BL_. According to the catch profile, the hake population retained by any design with CD_S_ would mostly be that above its MCRS, due to the release of undersized individuals. Here, we highlight that contrary to diamond meshes, where all mesh bars are under tension due to the forces acting on the gear, for square meshes tension is present only in the two longitudinal mesh bars [34], which favors mesh shape distortion outwards during an escape attempt. In our case, this may have been further facilitated due to differences in mesh material as, CD_S_ was constructed of polyethylene, a material less resistant to deformation than polysteel used in CD_D_ [52]. Besides, *L*50 of hake for CD_D_ showed to be low (15.68 (CI: 12.47–17.51)) when compared to results reported by Alzorriz et al. [19] (20.29 (CI: 17.64–24.08)) who used a diamond mesh size of 75.80 mm. Apart from mesh size, other factors such as catch size [31, 32, 79], netting orientation and twine thickness [29], or the number of meshes in the circumference [28] can affect codend size selectivity because they can alter codend shape. Although several characteristics of the codend used by Alzorriz et al. [19] and the used in this study were similar (mesh size and twine thickness), other differed. For example, Alzorriz et al. [19] made experimental trials on a fishing vessel, used a larger trawl, had longer towing times and probably, bigger catches. Although we cannot explain the differences found between these studies with certainty, we speculate that the differences in the experimental design mentioned may be the cause of the differences found. Conversely to hake, the delta plots in the treatment tree for blue whiting showed that any gear design that included an SMP at the bottom panel of the trawl increased the escape probability for this species (SMP_BS_ + CD_D_, SMP_BL_ + CD_D_, SMP_BS_ + CD_S_ or SMP_BL_ + CD_S_). Similar to other gadoids (e.g. haddock (*Melanogrammus aeglefinus*) or whiting (*Merlangius merlangus*)), which have a vertical preference of swimming in the upper part of the trawl [80], blue whiting showed higher escape probability through the SMP_TS_ and SMP_TL_ together with any codend design, including a fraction of commercial-size individuals. These results agree with previously reported data on the suitability of SMP designs placed at the top panel to release non-target blue whiting in trawl gears [62, 81, 82].

Since the meshes in SMP_BL_ *PC* and CD_S_ *CC* were too big to rule out that some of the smallest hake and blue whiting individuals could have escaped through the cover meshes, we cannot conclude on the outcome for individuals below 20 cm for SMP_BS_ and SMP_BL_, and below 15 cm for CD_D_ and CD_S_. For hake, there is experimental data around the MCRS (both below and over 27 cm length), and therefore, the interpretation of the results for the sizes around MCRS can be trusted. In case of blue whiting, whose marketable size limit is 18 cm, the experimental data included in the analyses for SMP_BS_ and SMP_BL_ are over this size and therefore, the results around 18 cm should be interpreted with care.

Exploitation pattern indicators were also estimated for hake and blue whiting, providing quantitative information about the suitability of the gear for a specific fishing situation [83, 84]. The configurations analyzed in this study show different exploitation patterns for hake and blue whiting depending on the population scenario fished. These results highlighted potential strategies for fishing vessels operating in this area. Comparison of the exploitation indicators of the different gear between both species reveals that in some population scenarios fishing interest focused on hake may conflict with other target species with lower MCRS or minimum marketable size, such as blue whiting. However, the results in this study show that this mismatch could be resolved by taking advantage of differences in escape behavior between species. For instance, since only hake individuals are released by the SMP_BL_, blue whiting would almost exclusively be size-selected by meshes in the codend. For example, in scenario (a), around 45% of legal-size blue whiting are estimated to be retained with SMP_BL_ + CD_S_, opposite to SMP_TS_ + CD_S_ and SMP_TL_ + CD_S_ gear designs, which were respectively estimated to retain around 40% and 26% less blue whiting above its minimum marketable size. These low values, especially when using SMP_TL_, could be seen as poor capture efficiency for blue whiting, although non-desired catches of this species in some fisheries often respond to market preferences [7]. In the Cantabrian Sea fisheries, for example, from the year 2000 on the single bottom trawl métier targeting blue whiting practically disappeared as a consequence of increased pair trawl effort in the area [85, 86]. The preference for blue whiting in bottom trawls operating in the Bay of Biscay may be conditioned by the more efficient pair trawls in ICES 8c, which target blue whiting. Additionally, whereas pair trawlers return to port almost every 24 hours, bottom trawlers in the Bay of Biscay (8abd) return every 6 days, which may imply retaining blue whiting during the last couple of days of the cruise to ensure the fish quality and freshness required by the market.

So far, the effort invested on attempting to open new paths towards sustainable exploitation patterns in these fisheries by means of the use of supplementary selection devices (e.g., SMPs) has shown that avoiding unwanted catches without losing target catch remains a problem. The results in this study state that SMP_BL_ + CD_S_ can favor catch patterns for hake because most undersized hake can be released for the majority of population scenarios. However, our data also show that the strongest effect on the catches are obtained when a square mesh codend is used, suggesting that simple codend adjustments may provide the opportunity to improve the size selectivity for hake and blue whiting. Although fishermen are often reluctant to codend modifications, especially in mixed fisheries, bioeconomic simulations anticipated detrimental effects in the short-term for the Basque trawling fisheries under full compliance of the Landing Obligation as well as in mid-term when applying any kind of exemption or flexibility to the law (current situation) [87, 88]. Therefore, we believe that further research should prioritize codend size selectivity, and additional selection devices may be added once codend designs with good selective properties are achieved.

Finally, graphics are becoming increasingly important for scientists to effectively communicate their findings to broad audiences. We believe that the treatment trees used in this study greatly improves the readability and interpretation of selectivity results and therefore, may aid the identification of promising and compatible gear designs, thus helping the industry in the pursuit of individual catch goals. The exploitation pattern indicators proved to be the fastest measure to determine which gear design could represent a viable option for a case-study fishery and the traffic-light procedure implemented categorized multiple exploitation indicators, providing by overview easily understandable results for managers and stakeholders. We therefore find the approach used in this study a powerful tool to periodically evaluate the performance of fishing gears in different fisheries around the world, which could potentially support and speed up the decision-making process made by fishing commissions, states or stakeholders.
